# The Calcium-Sensing Receptor Is Involved in Follicle-Stimulating Hormone-Induced Cumulus Expansion in *in vitro* Cultured Porcine Cumulus-Oocyte Complexes

**DOI:** 10.3389/fcell.2021.625036

**Published:** 2021-05-20

**Authors:** Huage Liu, Dan Zhou, Cong Liu, Qingrui Zhuan, Yan Luo, Xianhong Mo, Xiangwei Fu, Yunpeng Hou

**Affiliations:** ^1^Institute of Reproductive Medicine, Nantong University, Nantong, China; ^2^State Key Laboratory of Agrobiotechnology, College of Biological Sciences, China Agricultural University, Beijing, China; ^3^Key Laboratory of Animal Genetics, Breeding and Reproduction, Ministry of Agriculture and National Engineering Laboratory for Animal Breeding, College of Animal Science and Technology, China Agricultural University, Beijing, China; ^4^School of Basic Medical Sciences, Wuhan University, Wuhan, China; ^5^College of Life Sciences, Chifeng University, Chifeng, China

**Keywords:** CASR, cumulus expansion, FSH, *in vitro* maturation, pig

## Abstract

The Calcium-Sensing Receptor (CASR) is a G protein-coupled receptor of the C family that reportedly promotes maturation of porcine oocytes. However, its role in cumulus expansion of cumulus-oocyte complexes (COCs) is not well known. This study was conducted to determine the role of CASR and potential mechanisms involved during *in vitro* maturation (IVM) of porcine COCs. After culture of COCs in follicle-stimulating hormone (FSH)-supplement maturation medium for 24 h, the time of breakdown of the germinal vesicle (GVBD), indicative of initiation of meiotic maturation, resulted in an increased (*p* < 0.05) *CASR* mRNA expression level in cumulus cells. Moreover, IVM of COCs in 10 μM of the CASR agonist NPS R-568 promoted (*p* < 0.05) cumulus expansion but only in FSH-containing medium. Conversely, 20 μM of the CASR inhibitor NPS2390 precluded cumulus expansion. We next tested the effect of the CASR agonist/inhibitor on the expression of cumulus expansion-related genes. The CASR agonist significantly upregulated the expression of hyaluronan acid synthase 2 (*HAS2*), whereas the CASR inhibitor downregulated the expression of all *HAS2*, prostaglandin-endoperoxide synthase 2 (*PTGS2*), and tumor necrosis factor a-induced protein 6 (*TNFAIP6*). Altogether, these results suggest that CASR activity is involved in FSH-stimulated porcine cumulus expansion.

## Introduction

During follicular development, mammalian oocytes undergo a series of important changes induced by the pre-ovulatory surge of gonadotropins (Eppig, 1996). The endogenous luteinizing hormone (LH) peak initiates the meiotic resumption of oocytes arrested in the dictyate stage of meiotic prophase. The meiosis resumption is defined by the occurrence of germinal vesicle breakdown and is accompanied by transformation of the cumulus oophorus surrounding the oocyte, known as “expansion” (Eppig, 1979b).

The components of *in vitro* maturation medium play an essential role in the degree of cumulus cell expansion and oocyte maturation (Qian et al., 2003; Appeltant et al., 2016). For instance, FSH enhances cumulus expansion during *in vitro* culture of canine (Lee et al., 2007) and mouse (Eppig, 1979a) cumulus-oocyte complexes (COCs). Epidermal growth factor (EGF) can also stimulate cumulus expansion *in vitro* in mouse (Downs, 1989; Boland and Gosden, 1994) and bovine (Lorenzo et al., 1994). Moreover, FSH (Nagyová et al., 1999) and EGF (Procházka et al., 2000; Jezová et al., 2001)-induced cumulus expansion correlated with the synthesis of hyaluronan by porcine cumulus cells and its accumulation. Notably, the expression of genes involved in the production of hyaluronic acid and its organization in the extracellular matrix, such as hyaluronan acid synthase 2 (*HAS2*), prostaglandin-endoperoxide synthase 2 (*PTGS2*), and tumor necrosis factor a-induced protein 6 (*TNFAIP6*), is increased preceding cumulus expansion in mouse COCs (Park et al., 2004).

Calcium (Ca^2+^), the most universal second messenger, is modulated through numerous cell-surface receptors to activate multiple cytoplasmic signaling proteins (Berridge et al., 2000). In this context, Ca^2+^ signaling pathways play crucial roles in gamete development and maturation as well as fertilization and early embryonic development (Wakai and Fissore, 2013). The Calcium-Sensing Receptor (CASR), a member of the G protein-coupled receptors, is an important regulator of (Ca^2+^)_0_ concentrations (Brown, 2013; Tyler Miller, 2013). Calcium-Sensing Receptor (CASR) activation in cells results in intracellular Ca^2+^ mobilization, regulation of intracellular cAMP levels and activation of multiple protein kinases (Ellinger, 2016). Studies indicate that expression of CASR has been detected in human, equine and porcine oocytes (Dell’Aquila et al., 2006; De Santis et al., 2009; Liu et al., 2015), and rat testicular tissue and sperm (Mendoza et al., 2012). Moreover, it was suggested that CASR participated in gonadotropin-induced porcine oocyte nuclear maturation (Liu et al., 2015), but its potential role in cumulus expansion was not determined. Therefore, the objective of this study was to investigate the effect of CASR on FSH-induced cumulus expansion and the consequent expression of expansion-related genes, namely *HAS2*, *PTGS2*, and *TNFAIP6*, in *in vitro* cultured porcine COCs.

## Materials and Methods

All chemicals for this study were purchased from Sigma Chemicals Co. (St. Louis, MO), unless otherwise stated. The present study was approved by the Institutional Animal Care and Use Committee of China Agricultural University.

### Cumulus Oocyte Complex (COC) Collection

Ovaries were collected from prepubertal Landrace gilts at a local slaughterhouse, transported to the laboratory within 2 h from slaughter and washed three times with 37°C 0.9% (w/v) NaCl containing 65 mg/l potassium penicillin G and 50 mg/l streptomycin sulfate. COCs were aspirated from antral follicles (3–8 mm diameter) with an 18-gauge needle fitted to a 10-ml disposable syringe. Aspirates were flushed with pre-warmed Tyrode’s medium (TLH) containing 0.1% (w/v) polyvinyl alcohol (PVA) (TLH-PVA) (Funahashi et al., 1997). Those with uniform cytoplasm and at least four layers of intact, compact cumulus cells were selected under a microscope (SZ61, Olympus, Tokyo, Japan).

### Immunofluorescence and Confocal Microscopy

Some available COCs were used immediately after collection for immunofluorescence studies. According to Liu et al. (2015), COCs were fixed in 4% paraformaldehyde for at least 30 min at room temperature and thoroughly washed three times. Then they were permeabilized in Dulbecco’s phosphate buffered saline (DPBS, Gibco, Grand Island, NY) containing 1% Triton X-100 for 1 h at 37°C and blocked in DPBS containing 2% BSA at 37°C for 30 min. COCs were incubated with anti-CASR primary antibody (sc-32181, Santa Cruz Biotechnology, Santa Cruz, CA, United States) diluted 1:25 in blocking buffer at 37°C for 2 h. After washing three times, samples were incubated with DyLight^TM^ 488-conjugated AffiniPure Rabbit anti-Goat IgG (Jackson ImmunoResearch, West Grove, PA) diluted 1:35 in blocking buffer at 37°C for 1 h (in the dark). Nuclear DNA was counterstained with DAPI (sc-24941, Santa Cruz Biotechnology, Santa Cruz, CA, United States) for 10 min. Then samples were mounted on glass slides and examined with a confocal laser-scanning microscope (FLUOVIEW FV1000, Olympus, Tokyo, Japan). The excitation lasers were set at 488 nm and 405 nm for green and blue fluorescence, respectively.

### Western Blot Analysis

According to previous study (Liu et al., 2015), total protein was extracted from 200 denuded oocytes and the corresponding cumulus cells immediately after collection. For protein extraction, samples were treated in 2 × Laemmli sample buffer and boiled for 10 min followed by cooling on ice. Total proteins were separated by SDS-PAGE and transferred to nitrocellulose filter membranes (0.45-μm pore size, Bio-Rad Laboratories, Richmond, CA, United States). The membrane was blocked in Tris-buffered saline Tween-20 (TBST, TBS with 0.05% Tween 20) containing 5% (w/v) non-fat dry milk for 2 h, and then incubated with the anti-CASR (1:300 dilution, sc32181, Santa Cruz Biotechnology, Santa Cruz, CA) or anti-β actin (1:1,000 dilution, TA-09, ZSGB, Beijing, China) primary antibodies in TBST containing 5% (w/v) non-fat dry milk for 2 h at room temperature. After three 10-min washes in TBST, membranes were incubated with horseradish peroxidase (HRP)-conjugated donkey anti-goat IgG (1:10,000) and goat anti-rabbit IgG (1:2,000) secondary antibodies for CASR and ACTIN, respectively, for 1 h at room temperature. Immunoreactive signals were detected with an enhanced chemiluminescence kit (Merck Chemical Co., Darmstadt, Germany) according to the manufacturer’s instructions.

### *In vitro* Maturation (IVM)

The basic maturation medium was tissue culture medium 199 (TCM199, Gibco, Grand Island, NY, United States) with Earle’s salts supplemented with 0.57 mM cysteine, 0.91 mM sodium pyruvate, and 0.1% (w/v) PVA (Yuan and Krisher, 2010). According to the study by Liu et al. (2015), either the CASR agonist NPS R-568 (Tocris Bioscience Bristol, Bristol, United Kingdom) or antagonist NPS2390 was added to the basic medium supplemented with or without 0.01 U/ml FSH (Sioux Biochemical, Sioux Center, IA). The treatment was as follows: (1) Basic medium (FSH-free); (2) Addition of CASR agonist (5 or 10 μM) to basic medium (FSH-free + A); (3) Addition of CASR antagonist (10 or 20 μM) to basic medium (FSH-free + I); (4) Addition of 0.01 U/ml FSH to basic medium (FSH, control group); (5) Addition of CASR agonist (5 or 10 μM) to (4) (FSH + A); (6) Addition of CASR antagonist (10 or 20 μM) to (4) (FSH + I).

Groups of 80–100 COCs were washed three times with pre-equilibrated IVM medium and cultured in 500 μl IVM medium at 39°C in an atmosphere of 5% CO_2_ and saturated humidity. After incubation for 24 h, cumulus cells were removed by gently pipetting in TLH-PVA medium containing 0.1% (w/v) hyaluronidase and then washed with TLH. COCs in each group were used to determine the degree of cumulus cell expansion and gene expression.

After the cumulus cells and oocytes were completely separated, the cumulus cells were centrifuged for 5 min (800 g), washed twice with phosphate-buffered saline (PBS), and then plated into a 24-well plate. The cells were cultured in DMEM/F12 (Gibco), 1% penicillin and streptomycin (HyClone) and 10% fetal bovine serum (Biological Industries, Kibbutz Beit Haemek) and placed in a 38.5°C incubator with 5% CO_2_.

### RNA Interference

Cumulus cells cultured in 24-well plates were transfected with small interfering RNA (siRNA) against CASR (CASR-siRNA) or negative control siRNA (NC-siRNA) using Lipofectamine RNAiMAX transfection reagent (Thermo Fisher Scientific) according to the manufacturer’s instructions. Briefly, 10 μM negative control siRNA (NC-siRNA) or CASR siRNA (CASR-siRNA) were diluted and mixed with Lipofectamine RNAiMAX Reagent. After mixing and incubation for 20 min, the transfection mixture was added to the cells cultured in DMEM/F12. The siRNAs were synthetized by GenePharma (GenePharma). The siRNA sequences are shown in Supplementary Table 1.

### RNA Isolation, Reverse Transcription PCR (RT-PCR) and Quantitative Reverse Transcription PCR (qRT-PCR)

Total RNA was isolated from 100 COCs (for measuring *HAS2*, *PTGS2* and *TNFAIP6*) and the cumulus cells of 100 COCs (for measuring *CASR*) using TRIzol reagent (Invitrogen, Carlsbad, CA, United States). RNA concentration and purity were quantified using a Nanodrop ND-1000 Spectrophotometer (Biolab, Scoresby, Victoria, Australia). After isolation, RNA from each treatment group was reverse transcribed into cDNA (High-Capacity cDNA RT kit, Applied Biosystems, Foster City, CA, United States).

RT-PCR for *CASR* was performed according to the manufacturer’s instructions (Tiangen Biotech, Beijing, China) and fragments generated were visualized by gel electrophoresis. qRT-PCR for *CASR* and expansion-related genes expression was performed by adding 1 μl of cDNA to a mixture of SYBR premix qPCR SuperMix (Qiagen, Valencia, CA).

### RNA Isolation, Reverse Transcription PCR (RT-PCR) and Quantitative Reverse Transcription PCR (qRT-PCR)

Total RNA was isolated from 100 COCs (for measuring *HAS2*, *PTGS2* and *TNFAIP6*) and the cumulus cells of 100 COCs (for measuring *CASR*) using TRIzol reagent (Invitrogen, Carlsbad, CA, United States). RNA concentration and purity were quantified using a Nanodrop ND-1000 Spectrophotometer (Biolab, Scoresby, VIC, Australia). After isolation, RNA from each treatment group was reverse transcribed into cDNA (High-Capacity cDNA RT kit, Applied Biosystems, Foster City, CA, United States).

RT-PCR for *CASR* was performed according to the manufacturer’s instructions (Tiangen Biotech, Beijing, China) and fragments generated were visualized by gel electrophoresis. qRT-PCR for *CASR* and expansion-related genes expression was performed by adding 1 μl of cDNA to a mixture of SYBR premix qPCR SuperMix (Qiagen, Valencia, CA, United States), forward and reverse primers (10 μM) and RNase-free water, in a final volume of 20 μl using an ABI 7,500 real-time PCR instrument (Applied Biosystems, Foster City, CA, United States). Cycling conditions were: 94°C for 30 s; 40 cycles at 94°C for 5 s and 60°C for 34 s. The mRNA level of each sample was normalized to *ACTIN* mRNA level. Relative transcriptional levels of target genes were calculated using the 2^–△△Ct^ method (Livak and Schmittgen, 2001). PCR primers used for real-time PCR are listed in Table 1.

**TABLE 1:** Primers used for real-time PCR.

**Gene transcript**	**GenBank accession number**	**Primers**	**Amplicon length (bp)**	**T_*an*_ (°C)**
*CASR*	NM_001278748	F: 5′-GCAGGATAAGCA ATAGCTCCA-3′ R: 5′-AAAGTTTAAGTG CCGTAGGTG-3′	263	57
*HAS2*	NM_214053	F: 5′-GAAGTCATGGG CAGGGACAATTC-3′ R: 5′-TGGCAGGCCC TTTCTATGTTA-3′	407	55
*PTGS2*	NM_214321	F:5′-TCGACCAGAGCA GAGAGATGAGAT-3′ R: 5′-ACCATAGAGC GCTTCTAACTCTGC-3′	260	55
*TNFAIP6*	NM_001159607	F: 5′-GAAGCACGGTC GGGCAAG-3′ R: 5′-CATCCACCCAG CAGCACAG-3′	141	57
*ACTIN*	Q6QAQ1	F: 5′-GCTTCTAGGCG GACTGTTAG-3′ R: 5′-ACCTTCACCGTT CCAGTTTT-3′	189	57

*CASR, Calcium-sensing receptor; HAS2, hyaluronan synthase 2; PTGS2, prostaglandin-endoperoxide synthase 2; TNFAIP6, tumor necrosis factor a-induced protein 6; ACTIN, beta actin.*

### Evaluation of Cumulus Expansion

At the end of incubation after 24 h, the degree of cumulus cell expansion was assessed by light microscopy. For this purpose, digital images of COCs on a stage micrometer were captured at 400× magnification (Nikon, Tokyo, Japan). Briefly, the diameter of each COC was calculated by averaging the largest and smallest diameters (except for a few COCs in which diameters could not be determined from the images) (Koike et al., 2010) using Image J analysis (Supplementary Figure 1).

### Statistical Analysis

The data were analyzed using Kruskal-Wallis test, Mann-Whitney *U*-test and independent *t*-test from SPSS (version 17, Chicago, IL, United States), except for the qRT-PCR results of CASR inhibitor treatment using two-tailed *t*-tests in Microsoft Excel. *p* < 0.05 was considered statistically significant.

## Results

### Expression and Localization of CASR in Porcine Cumulus Cells

**FIGURE 1:**
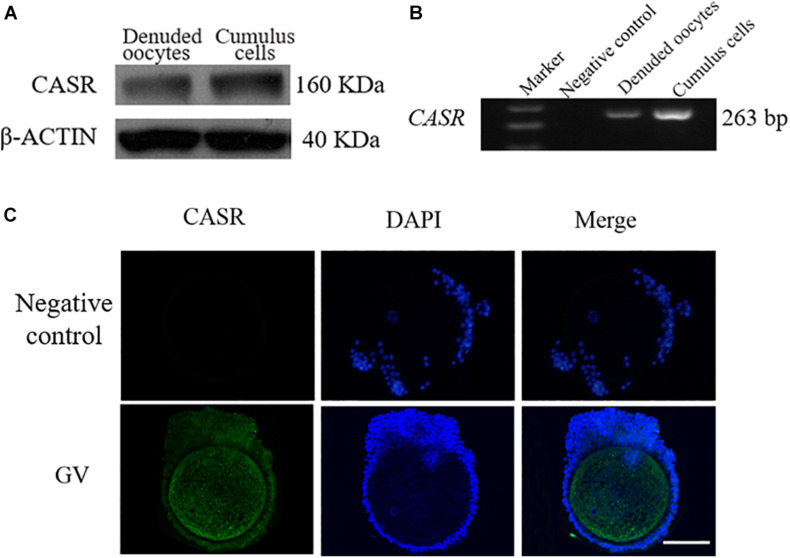
Expression and localization of CASR in porcine cumulus cells. Cumulus-oocyte complexes (COCs) or oocytes in the germinal vesicle (GV) stage (intact nucleus) were used immediately after collection. Identification of CASR protein **(A)** and mRNA **(B)** in porcine cumulus cells and denuded oocytes; negative control without cDNA. **(C)** Immunofluorescence results showed that CASR protein (green) was expressed in cumulus cells of porcine COCs. Chromatin was stained with DAPI (blue). Negative controls show no staining for CASR. In the negative control group, the first antibody was replaced by 2% BSA, with the other steps being the same. Scale bar = 50 μm. CASR, Calcium-Sensing Receptor.

*CASR* protein (a single ∼160 kDa protein band, Figure 1A) and *m*RNA (expected length 263 bp, Figure 1B) were detected in porcine cumulus cells and denuded oocytes. Immunofluorescence results showed that in addition to localizing in oocytes, CASR was expressed in cumulus cells of porcine COCs (Figure 1C).

### Expression of *CASR* mRNA in Cumulus Cells

**FIGURE 2:**
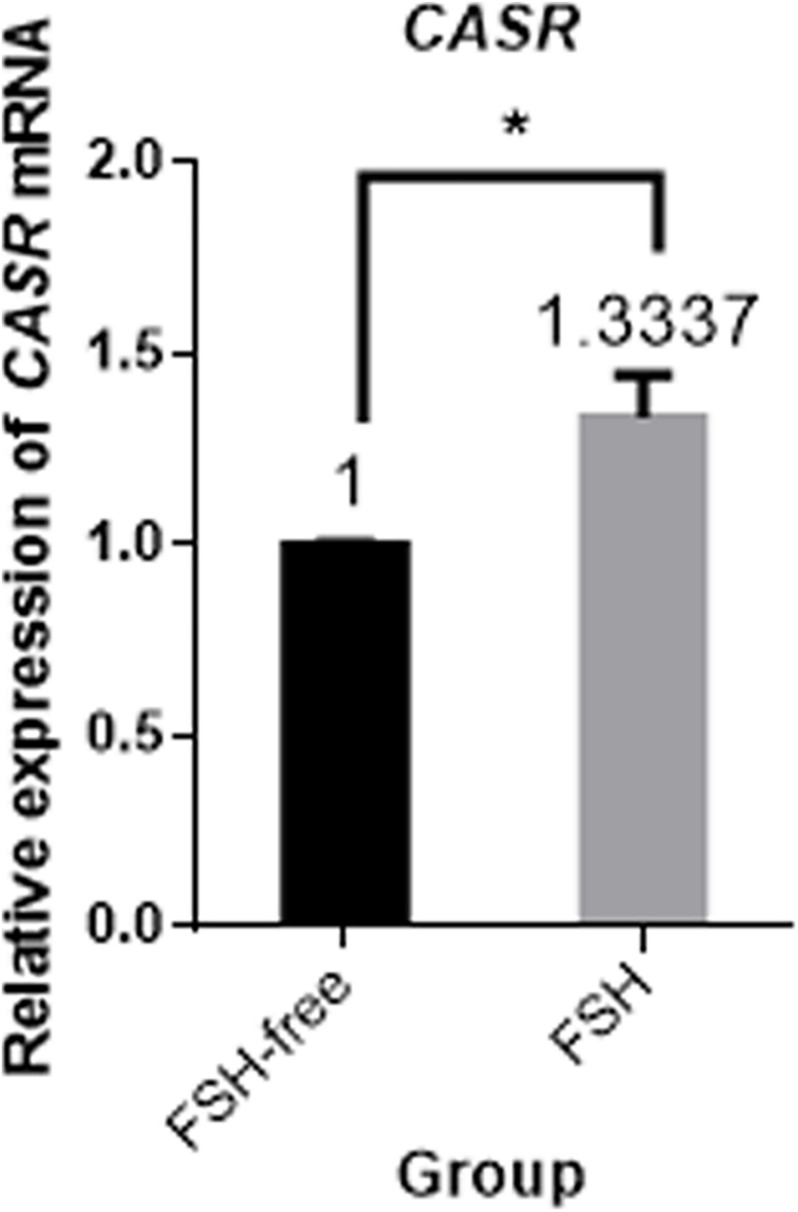
Relative expression of *CASR* mRNA in porcine cumulus cells from COCs matured in the presence or absence of FSH for 24 h by qRT-PCR. Data are presented as means ± SEM (*p < 0.05) from three independent experiments. (*) indicates significance between groups. Significance was determined by independent *t*-test. CASR, Calcium-Sensing Receptor.

Next, the effect of IVM with and without FSH on CASR expression was tested. Culture of COCs for 24 h in FSH-containing medium upregulated the expression of *CASR* mRNA in cumulus cells (*p* < 0.05; Figure 2).

### Effect of CASR Activity on Cumulus Expansion

**FIGURE 3:**
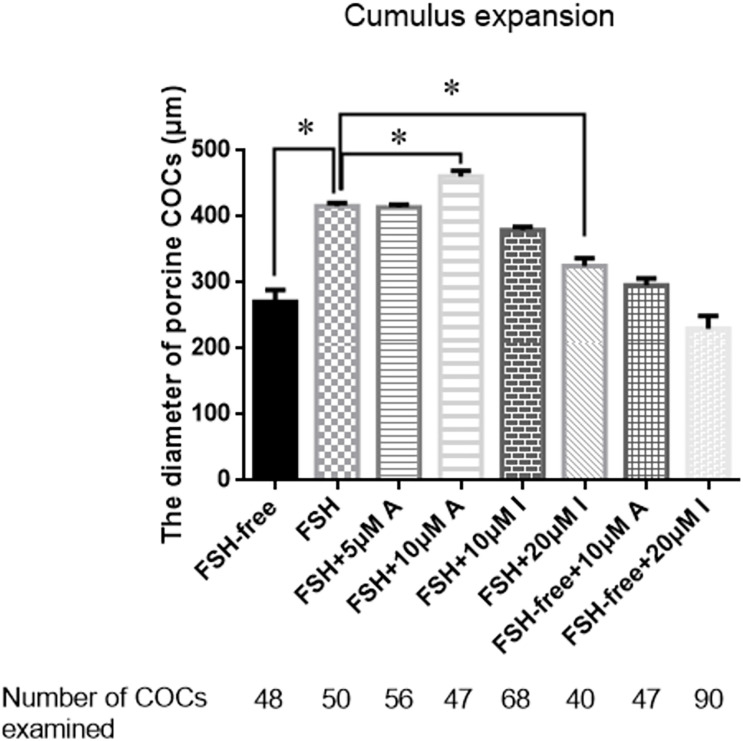
Effects of CASR activation or inhibition on cumulus cell expansion during IVM of porcine COCs. COCs were cultured in base IVM medium supplemented with FSH (0.01 U/ml), and/or the CASR agonist NPSR-568 (5 or 10 μM, A) and/or the CASR inhibitor NPS2390 (10 or 20 μM, I) for 24 h. FSH group was taken as a unit. Statistically significant differences are examined by independent *t*-test. Data are expressed as mean ± SEM from three independent experiments (*p < 0.05). CASR, Calcium-Sensing Receptor; A, CASR agonist NPSR-568; I, CASR inhibitor NPS2390.

To determine whether CASR was involved in cumulus expansion *in vitro* and, if so, whether the presence of FSH was critical, either the CASR agonist NPS R-568 or inhibitor NPS2390 were added to the maturation medium in different treatments. Notably, absence of FSH precluded adequate cumulus cell expansion to a level lower than that observed in the presence of FSH (*p* < 0.05; Figure 3). Adding either 5 μM CASR agonist or 10 μM CASR inhibitor to FSH-containing medium, the relative level of cumulus expansion had no significant variation, as shown in Figure 3 (*p* > 0.05). Further, addition of 10 μM CASR agonist in FSH-containing medium promoted cumulus expansion (*p* < 0.05). Conversely, addition of 20 μM CASR inhibitor significantly prevented cumulus expansion of COCs cultured in FSH-containing medium (*p* < 0.05). However, under the premise of free of FSH in the medium, compared to the FSH-free group, neither 10 μM CASR agonist nor 20 μM inhibitor had an effect on cumulus expansion when added in FSH-free medium (Figure 3). Following from these results, 10 μM CASR agonist and 20 μM CASR inhibitor were chosen for the next set of experiments. In order to identify the observed effect mediated specifically by CASR, the experiment with combination of the agonist and inhibitor was performed. The result showed that the effect of the CASR agonist observed with the COCs was significantly inhibited by the CASR inhibitor (Supplementary Figure 2).

**FIGURE 4:**
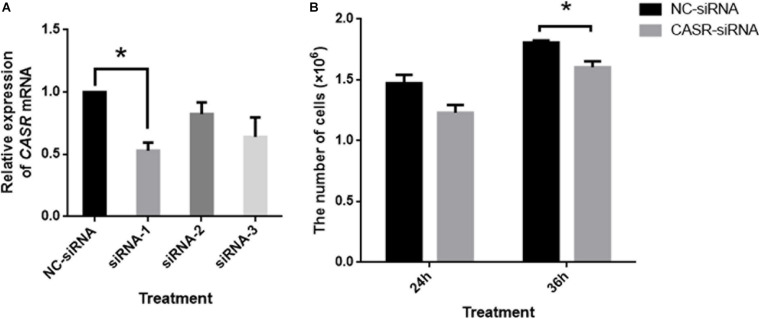
Effects of CASR activation inhibited by siRNA on cumulus cell proliferation. Cumulus cells treated by negative control siRNA (NC-siRNA) and three kinds of small interfering RNA against CASR (CASR-siRNA) were collected. **(A)** The relative *CASR* mRNA expression levels in the CASR-siRNA- and NC-siRNA-treated groups were determined by RT-qPCR. *ACTIN* mRNA was used as internal control. **(B)** The proliferation ability of cumulus cells was tested to reflect the cumulus expansion after CASR was inhibited by siRNA for 24 h and 36 h. CASR: Calcium-Sensing Receptor. The data represent means ± SEM (*p < 0.05) from at least two independent experiments. (*) indicates significance between groups. Significance was determined by Student-Newman-Keuls **(A)** or two-tailed *t*-tests **(B)**.

Additionally, the mRNA expression of CASR in porcine cumulus cells was inhibited with small interfering RNA (siRNA). The results showed that small interfering RNA against CASR (CASR-siRNA) reduced the expression of CASR mRNA, especially for the siRNA-1. Then we observed the proliferation ability of cumulus cells to reflect the cumulus expansion after CASR was inhibited by siRNA. The result showed that the number of cells had no significant variation between control group and CASR-siRNA group after 24 h (*p* > 0.05), however, the number of cells in CASR-siRNA group was significantly lower than that in control group when treated for 36 h (*p* < 0.05; Figure 4).

### Effect of CASR Activity on the Expression of Genes Involved in Cumulus Expansion

**FIGURE 5:**
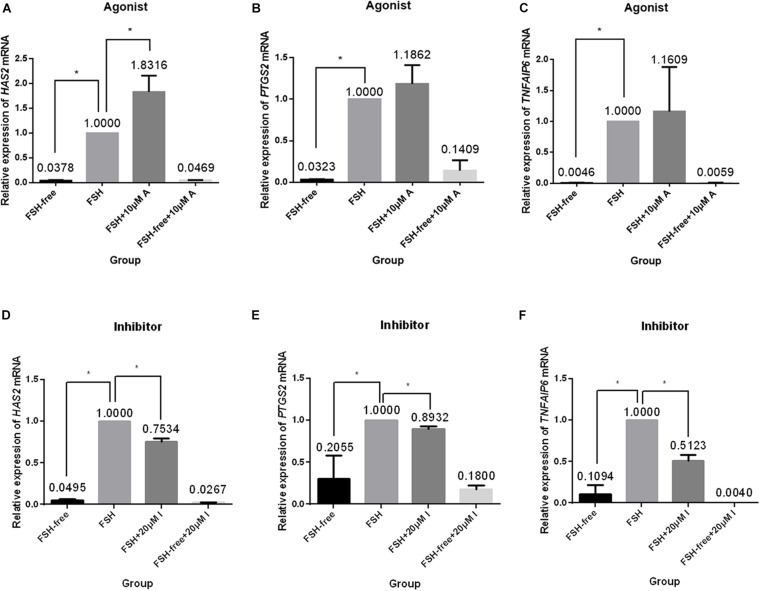
Relative expression of genes related to cumulus cell expansion in porcine COCs cultured under different conditions. COCs were cultured in medium supplemented with the CASR agonist NPSR-568 (10 μM, **A–C**) or inhibitor NPS2390 (20 μM, **D–F**) in the presence or absence of FSH for 24 h. *ACTIN* mRNA was used as internal control. *HAS2*: hyaluronan synthase 2; *PTGS2:* prostaglandin-endoperoxide synthase 2; *TNFAIP6:* tumor necrosis factor a-induced protein 6; CASR: Calcium-Sensing Receptor. The data represent means ± SEM (*p < 0.05) from at least two independent experiments. (*) indicates significance between groups; (ns) indicates no significance between groups. Significance was determined by Kruskal-Wallis test and Mann-Whitney *U*-test **(A–C)** or two-tailed *t*-tests **(E,F)**.

We next investigated the relative expression levels of expansion-related genes (*HAS2*, *PTGS2*, and *TNFAIP6*), which are known to participate or regulate cumulus expansion, in COCs cultured in the presence or absence of FSH with or without the addition of the CASR agonist (10 μM) or inhibitor (20 μM). Following 24 h of culture, the presence of the CASR agonist increased the expression of *HAS2* (*p* < 0.05; Figure 5A), without affecting expression levels of *PTGS2* and *TNFAIP6* (Figures 5B,C), but only in FSH-containing medium. Conversely, compared with the control group (FSH group), addition of the CASR inhibitor to the IVM medium significantly downregulated expression levels of all three genes (*p* < 0.05; Figures 5D–F). However, the CASR agonist or inhibitor had no effect in the level of expression of these three genes in FSH-free groups (Figure 5).

## Discussion

The study aimed to investigate the expression and influence of the CASR on cumulus expansion during maturation of porcine COCs. We demonstrate that CASR is expressed both at the mRNA and protein levels in porcine cumulus cells, in addition to oocytes (Liu et al., 2015). Expression was previously reported in equine and human COCs yielding a single 130 kDa protein and a 130/120 kDa protein doublet for oocytes and cumulus cells, respectively (Dell’Aquila et al., 2006; De Santis et al., 2009). In contrast, a single band of 160 kDa was detected in porcine cumulus cells, which is consistent with a previous study (Liu et al., 2015). This may reflect different levels of protein glycosylation among different species (Bai et al., 1996; Brown and MacLeod, 2001; Bai, 2004).

Importantly, we showed that the activity of the CASR contributed to FSH-stimulated cumulus expansion during initiation of meiotic resumption. A previous study had shown that CASR activity was pivotal for oocyte maturation by mediating the effects of gonadotropins (Liu et al., 2015); however, the potential effects of CASR on cumulus cells were not investigated. Given that FSH promotes cumulus expansion during IVM in canine (Lee et al., 2007), pig (Singh et al., 1993), and rat (Phillips and Dekel, 1982), we hypothesized that CASR should contribute to this effect also. Therefore, we first showed that CASR transcript levels were upregulated in cumulus cells when COCs were cultured in FSH-containing medium. Moreover, when porcine COCs were cultured in the presence of a CASR protein agonist (NPS R-568) or inhibitor (NPS2390) cumulus expansion was significantly enhanced or inhibited, respectively. Interestingly, the stimulatory effects of the CASR agonist were not observed in medium devoid of FSH. Altogether, these results suggest that the CASR depends upon FSH to promote cumulus expansion.

We next investigated changes in expression of genes related to hyaluronic acid synthesis and maintenance. Interestingly, the presence of a CASR activator in the COC culture medium significantly upregulated the expression of *HAS2*, a gene regulated by gonadotropins and required for cumulus expansion (Kimura et al., 2002; Nagyova et al., 2012). *HAS2* gene encodes hyaluronan synthase enzyme which is involved in synthesis of hyaluronan. FSH stimulated cumulus expansion correlated with the synthesis of hyaluronan by porcine cumulus cells (Nagyová et al., 1999), and upregulated CASR expression. Then, CASR may be involved in FSH-induced cumulus expansion by increasing the synthesis of hyaluronan via the upregulated expression of *HAS2*. However, CASR activator supplementation did not change *PTGS2* and *TNFAIP6* expressions. It will be difficult to propose at this moment that whether *PTGS2* or *TNFAIP6* expression is regulated by some other mechanism (Chaubey et al., 2018). Moreover, the presence of the CASR inhibitor in the medium also significantly downregulated genes related to hyaluronic acid synthesis and cumulus expansion. Notably, as above, the effects of the CASR activator/inhibitor were only observed in FSH-containing medium. These data may provide a link between the effects of FSH-CASR-mediated cumulus expansion during maturation of porcine COCs. As gap junctional communications may play a role in cumulus expansion and gap junction (GJ) inhibitor Carbenoxolone (CBX) reduced the extent of cumulus expansion during the first 20 h of IVM (Appeltant et al., 2015), CASR may affect cumulus expansion through gap junction in the current study. Receptor activity-modifying proteins (RAMPs) is necessary to become the immature CASR into fully glycosylated prior to delivery to the plasma membrane (Bouschet et al., 2005). The mature CASR in the plasma membrane may be used as a marker of oocyte maturation. Given that CASR may have some interactions between cumulus cells and oocytes through gap junction, it leads to the redistribution of CASR to the plasma membrane in oocytes and further contributes to oocyte maturation. Indeed, activation of the mitogen activated protein kinase (MAPK) pathway in cumulus cells is essential for cumulus expansion of FSH-primed mouse COCs (Su et al., 2002). This pathway is also important for the expression of cumulus expansion-related genes during gonadotropin-induced maturation of porcine oocytes (Yamashita et al., 2009; Prochazka et al., 2012) as well as for FSH-induced cumulus expansion of mouse COCs (Diaz et al., 2006). Therefore, we hypothesize that CASR may affect FSH-stimulated porcine cumulus expansion through a MAPK signaling pathway in cumulus cells. In turn, MAPK may stimulate expression of EGF-like factors which impact on cumulus expansion and oocyte maturation (Yamashita et al., 2009). However, these hypotheses require further investigation.

## Conclusion

Our results support a role for CASR during cumulus expansion in porcine COCs, which can be regulated by FSH and promotes FSH-stimulated cumulus expansion. Whether CASR can act as a potent regulator of cumulus expansion requires further study.

## Data Availability Statement

The raw data supporting the conclusions of this article will be made available by the authors, without undue reservation.

## Ethics Statement

The animal study was reviewed and approved by the present study was approved by the Institutional Animal Care and Use Committee of China Agricultural University.

## Author Contributions

HL and YH designed the study, conducted the experiments, interpreted the results, and drafted the manuscript. DZ, QZ, and YL conducted the part of the experiments. CL and XM provided the part of the idea. XF contributed to analysis and interpreted the results. All authors contributed to revise the manuscript.

## Conflict of Interest

The authors declare that the research was conducted in the absence of any commercial or financial relationships that could be construed as a potential conflict of interest.
