# Supplementary material for: The Calcium-Sensing Receptor Is Involved in Follicle-Stimulating Hormone-Induced Cumulus Expansion in in vitro Cultured Porcine Cumulus-Oocyte Complexes
Source: Front Cell Dev Biol. 2021 May 20;9:625036. doi: 10.3389/fcell.2021.625036 (PMC8173154; doi:10.3389/fcell.2021.625036)
Supplement: Supplementary file 1 [file Data_Sheet_1.pdf]

Supplementary Materials

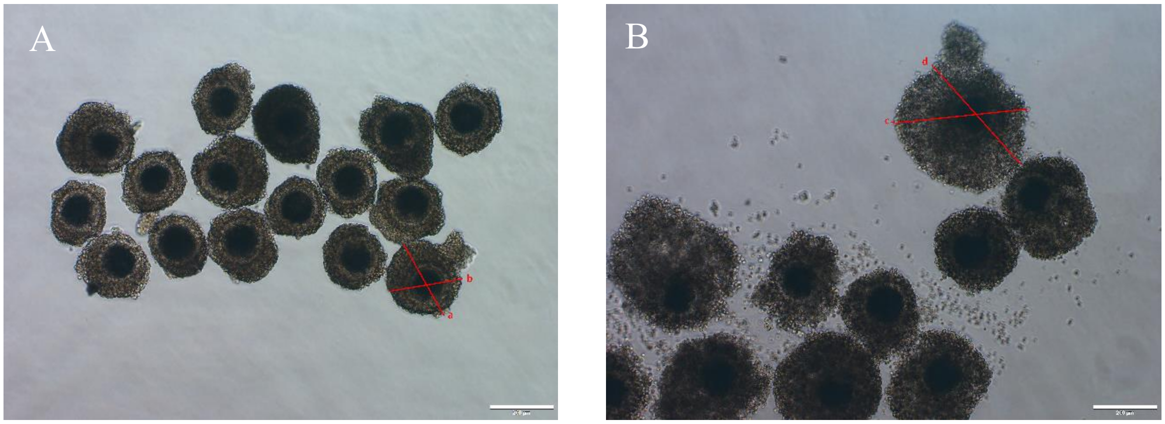

FIGURE S1 Representative images to illustrate evaluation of cumulus expansion. Diameters for each cumulus-oocyte complex were determined by averaging the largest and smallest diameter (a,b or c,d) using Image J analysis. Immediately following COC collection (A). After a 24-h maturation period (B). Then the change of diameter after COC expansion was calculated: [(c + d) / 2] / [(a + b) / 2]. The relative fold change was obtained by dividing the mean value of change in the diameter of total COCs in treatment group by that of COCs in control group. Scale bar = 200 μm.

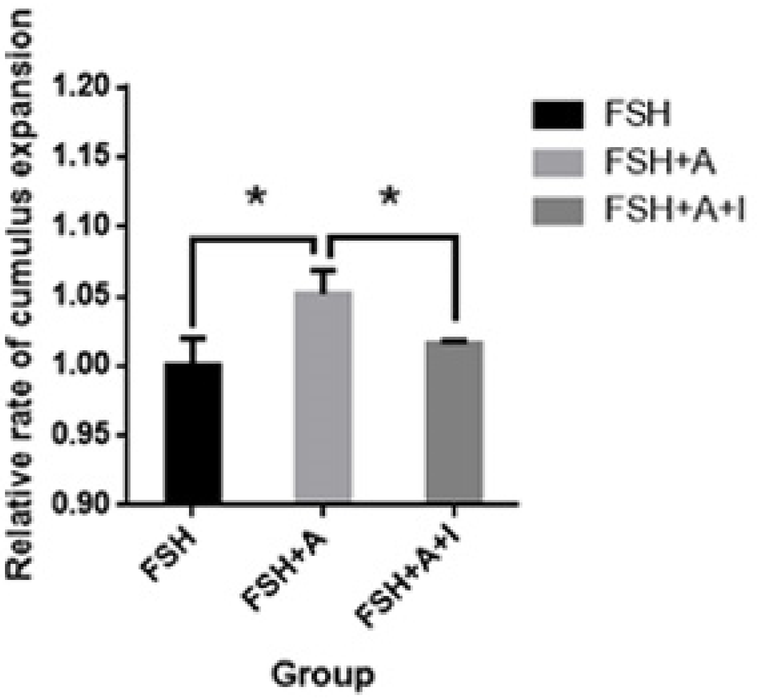

FIGURE S2 Relative cumulus expansion during IVM of porcine COCs. COCs were cultured in base IVM medium supplemented with FSH (0.01 U/ml), and the CASR agonist NPSR-568 (10 μM, A) and/or the CASR inhibitor NPS2390 (20 μM, I) for 24 h (FSH: n=50 COCs； FSH+A: n=45 COCs； FSH+A+I: n=47 COCs). *$p < .05$.

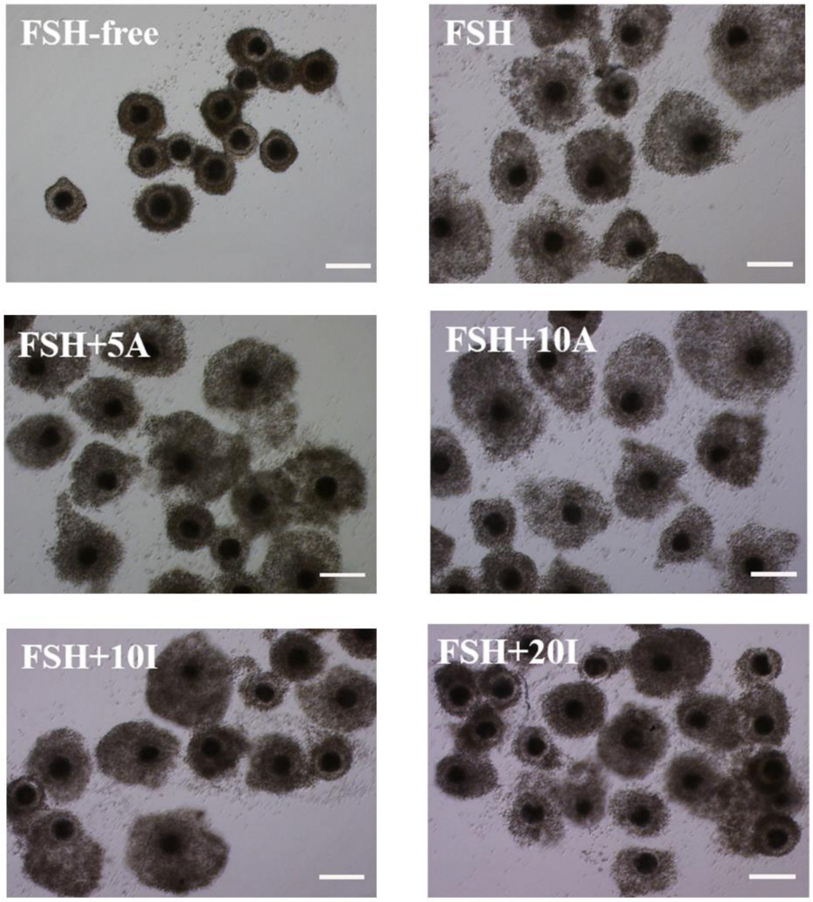

FIGURE S3 Representative images of cumulus expansion during IVM of porcine COCs. COCs were cultured in base IVM medium supplemented with FSH (0.01 U/ml), and/or the CASR agonist NPSR-568 (5 μM or 10 μM, A) and/or the CASR inhibitor NPS2390 (10 μM or 20 μM, I) for 24 h. Images were taken at the same magnification. Scale bar = 200 μm.

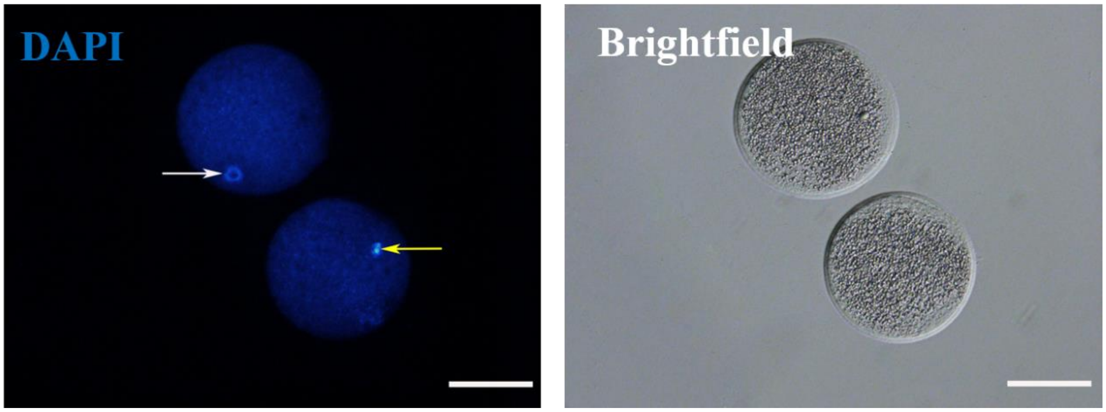

FIGURE S4 Representative images of GV and GVBD stage oocytes during IVM of porcine COCs. GV oocyte, germinal vesicle (white arrow); GVBD oocyte, germinal vesicle breakdown and chromosome condensation (yellow arrow). Scale bar = 50 μm.

Table S1 siRNA sequences used for RNA inference.

**Gene**	Primers
Negative control-siRNA	Sense: 5´-UUCUCCGAACGUGUCACGUTT-3´
	Antisense: 5´-ACGUGACACGUUCGGAGAATT-3´
CASR-siRNA-1	Sense: 5´-GCAGACUCCUCAGCAACAATT-3´
	Antisense: 5´-UUGUUGCUGAGGAGUCUGCTT-3´
CASR-siRNA-2	Sense :5´-GCUCAUGCCCUGCAAGAUATT-3´
	Antisense: 5´-UAUCUUGCAGGGCAUGAGCTT-3´
CASR-siRNA-3	Sense: 5´-GCAAGUGCCUGUGAUAAAUTT-3´
	Antisense: 5´-AUUUAUCACAGGCACUUGCTT-3´

CASR, Calcium-sensing receptor.

Table S2. Effect of CASR on germinal vesicle breakdown of oocytes.

Treatment	No. of oocytes	No. of GVBD (%)
FSH-free	53	20 (37.68±1.77)[a]
FSH	98	43 (55.46±1.23)[c]
FSH+5A	72	53 (56.76±0.51)[c]
FSH+10A	77	49 (63.63±0.27)[d]
FSH+10I	56	25 (45.31±2.70)[b]
FSH+20I	80	29 (34.87±1.98)[a]

GVBD: germinal vesicle breakdown.

COCs were cultured in base IVM medium supplemented with FSH (0.01 U/ml), and/or the CASR agonist NPSR-568 (5 μM or 10 μM, A) and/or the CASR inhibitor NPS2390 (10 μM or 20 μM, I) for 24 h.

[abc]Values within a column with different superscripts are significantly different ($P < 0.05$).
